# *HMGA2* regulates fear and growth: Canine GWAS and functional evidence

**DOI:** 10.1016/j.isci.2026.116429

**Published:** 2026-06-17

**Authors:** Yun Yu, Chao Li, Ye Liu, Yinyu Su, Xuebin Wang, James A. Serpell, Shurun Zhang, Jinxue Ruan, Yanhu Liu, Ya-Ping Zhang

**Affiliations:** 1 State Key Laboratory of Genetic Evolution & Animal Models and Yunnan Key Laboratory of Molecular Biology of Domestic Animals, Kunming Institute of Zoology, Chinese Academy of Sciences, Kunming 650201, China; 2 State Key Laboratory for Conservation and Utilization of Bio-resources, Yunnan University, Kunming 650091, China; 3 Key Laboratory of Agricultural Animal Genetics, Breeding and Reproduction of Ministry of Education & Key Lab of Swine Genetics and Breeding of Ministry of Agriculture and Rural Affairs, Huazhong Agricultural University, Wuhan 430070, P.R. China; 4 School of Veterinary Medicine, University of Pennsylvania, 3900 Delancey Street, Philadelphia, PA 19104, USA; 5 KIZ-CUHK Joint Laboratory of Bioresources and Molecular Research in Common Diseases, Kunming Institute of Zoology, Chinese Academy of Sciences, Kunming 650223, China; 6 Kunming College of Life Science, University of Chinese Academy of Sciences, Kunming, Yunnan 650204, China

**Keywords:** canine behavior, genomics, behavioral neuroscience

## Abstract

Fear is an evolutionarily conserved survival mechanism. Fear behavior is strongly correlated with body size in dogs, the genetic basis of fear regulation independent of size-related traits remains unclear. Through cross-breed genome-wide association analysis, we identified association between *HMGA2* gene and fear traits. Our functional validation revealed that viral overexpression of *Hmga2* in the mouse basolateral amygdala attenuated fear memory formation independent of body weight, while *Hmga2* knockout (KO) mice exhibited enhanced fear memory. Transcriptomic analyses linked *Hmga2* to modulation of neurotransmitter activity, synaptic plasticity (including GABAergic signaling), and neurogenesis, with additional roles in early brain development revealed by embryonic KO studies. We also identified a mutation in an enhancer that can regulate *HMGA2* expression in human HEK293T cell line. Our findings establish *HMGA2* as a pleiotropic regulator of fear behavior decoupled from its effects on body size, resolving a long-standing confound in canine behavioral genetics.

## Highlights

• HMGA2 is a pleiotropic fear regulator dissociated from its role in body growth
• HMGA2 modulates fear memory via synaptic plasticity, and GABAergic signaling
• HMGA2 is a key regulator of early brain development

## Graphical abstract

**Figure:**
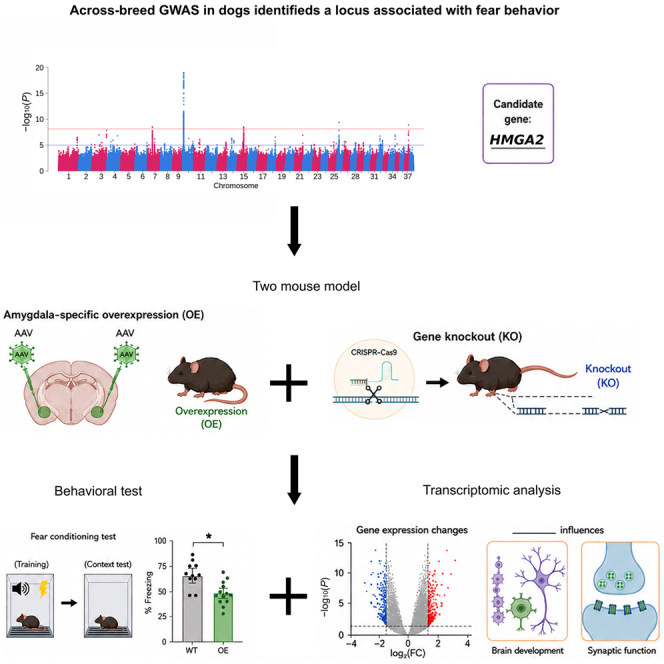

## Introduction

Fear is a normal response to threats and is a key to survival in vertebrate animals. Excessive or persistent fear or anxiety leads to mental disability. Problems related to fear are among the most prevalent forms of mental disorders. Symptoms characterized by excessive fear or anxiety include anxiety disorder and post-traumatic stress disorder (PTSD).^1^ Schizophrenia patients also have a high prevalence of abnormal fear.^2^

Dogs are a valuable resource for studying a variety of human diseases and disorders. Dogs exhibit naturally occurring fear behaviors that parallel human anxiety, making them a powerful model for dissecting the genetic architecture of fear. Uncovering genes underlying dog fear might provide valuable insights into the genetics of fear-related mental disorders. The Canine Behavior Assessment and Research Questionnaire (C-BARQ) is one of the most widely used dog behavior assessment tools.^3^^,^^4^ The C-BARQ assesses four types of fear or anxiety, being either social or non-social, which are measured by typical signs, such as avoiding eye contact, avoidance of the feared object, crouching, or cringing with tail lowered or tucked between the legs, whimpering or whining, freezing, and shaking or trembling, cowering, escaping, hiding, etc. Social fear includes fear of either unfamiliar humans (StrDirFear) or dogs (DogDirFear), while non-social fear (NonSocFear) generally involves fearful responses to sudden or loud noises (e.g., thunder), traffic, as well as unfamiliar objects and situations. The C-BARQ touch sensitivity factor (TouchSen) measures reactivity to touch and/or exposure to potentially painful procedures such as veterinary examinations.

Genome-wide association studies (GWASs) have revealed that genes associated with fear or anxiety in dogs overlap with human neuropsychiatric genes, respectively.^5^^,^^6^^,^^7^^,^^8^ Due to hundreds of years of selective breeding of a variety of breeds with varied characteristics, different dog breeds manifest considerable differences in behavior between breeds, and relatively high consistency within breeds, which enables the identification of genes underlying behavior by cross-breed genetic association studies with breed-defining behavioral phenotypes. Previous studies implied potential association between dog fear (TouchSen) or boldness and *HMGA2* gene.^9^^,^^10^ Shan et al.^11^ performed GWA analyses using American Kennel Club (AKC) functional breed categories as phenotypes and identified *MSRB3* and *CHL1* genes associated with fear in dogs and *MSRB3* is in fact the neighboring gene to *HMGA2*. However, these previous studies have either limited density of variations across the genome to identify candidate causal variants and genomic mechanisms, or lack sufficient functional validation and molecular mechanism exploration. Meanwhile, body size correlates with fear behavior in dog.^12^ When incorporating body size as a covariate, association between *HMGA2* and fear was not significant anymore. It remains unclear whether *HMGA2* exerts a direct pleiotropic effect on behavior, or whether its influence is indirect, mediated by body size. Specifically, smaller size may alter a dog’s social experiences such as increased vulnerability to bullying or different owner interactions, which in turn shape behavioral development.

While genetic associations provide critical leads, *in vivo* mechanistic validation is essential to move beyond genetic association and establish causal roles. The basolateral amygdala (BLA) is a conserved hub for fear processing across mammals, integrating sensory inputs and orchestrating fear responses.^13^ By manipulating *HMGA2* expression specifically within the BLA of mice, we can assess its role in fear-related behavior independent of its developmental effects on body size, thereby disentangling the pleiotropic effects observed in dogs. This technology has not been developed in dogs and also faces serious animal welfare and ethical issues.

In this study, we performed a cross-breed GWAS and identified *HMGA2* as a key gene underlying canine fear. Using *HMGA2* knockout (KO) and viral overexpression in mice, we demonstrated that *HMGA2* regulates fear memory independently of its effects on body size, resolving a long-standing pleiotropic confound. Transcriptomic analyses further showed that *HMGA2* regulates embryonic central nervous system (CNS) development, and postnatal neurotransmitter- and synapse-related pathways. Meanwhile, we revealed a variant within an enhancer that directly modulates *HMGA2* expression in HEK293T cell line.

## Results

### HMGA2 gene is associated with canine fear

**Figure 1:**
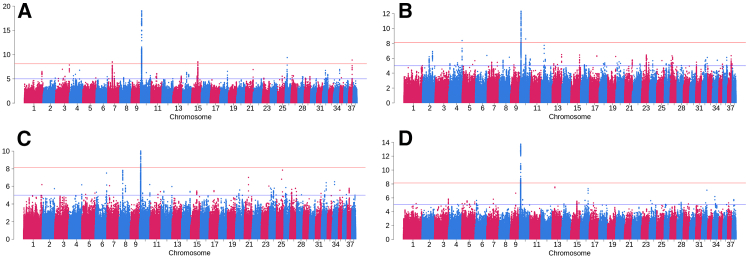
Manhattan plots of GWAS results (A–D) Manhattan plots showing the association of genome-wide variants with DogDirFear, referring to unfamiliar dog directed fear (A), StrDirFear, referring to unfamiliar person directed fear (B), NonSocFear, referring to non-social fear (C), TouchSen, referring to touch sensitivity (D). Bonferroni and suggestive significance thresholds are indicated with red and blue lines, respectively.

Single nucleotide polymorphisms (SNPs) and small insertions and deletions (InDels) derived from whole genome sequencing data from the Dog10K project was obtained.^14^ Behavior score data of 73,519 dogs from 393 breeds was obtained from the C-BARQ database at the University of Pennsylvania. Only the breeds shared between the Dog10K project and the C-BARQ dataset were kept for further GWAS analysis, specifically, 1,142 dogs from 223 breeds. Breed average scores for each of four types of fear, namely DogDirFear, StrDirFear, NonSocFear, and TouchSen, from the C-BARQ dataset were calculated and used as phenotypes in GWAS analyses. Only biallelic SNPs and InDels from autosomal chromosomes were kept for GWAS analysis. After quality control, 6,950,225 variants were left for the GWAS analysis. A common signal on chromosome 10 was obtained from the GWAS analyses of the four fear phenotypes (Figures 1A–1D). A correlation test between breed-average body weight (ranging from 5 pounds to 175 pounds, collected from American Kennel Club website) and fear was performed in these dogs, revealing that body weight significantly correlates with each of four types of fear in these dogs (supplemental 1, Figure S1). When incorporating body weight as a covariate in the GWAS model, the signal was not significant anymore (supplemental 1, Figures S2A–S2D). We speculate that this genomic region plays a pleiotropic role in regulating both body size and fear-related behavior. To further explore its pleiotropic role, we analyzed the signal on chromosome 10.

### Gain-of-function of Hmga2 in amygdala resulted in reduced fear memory

**Figure 2:**
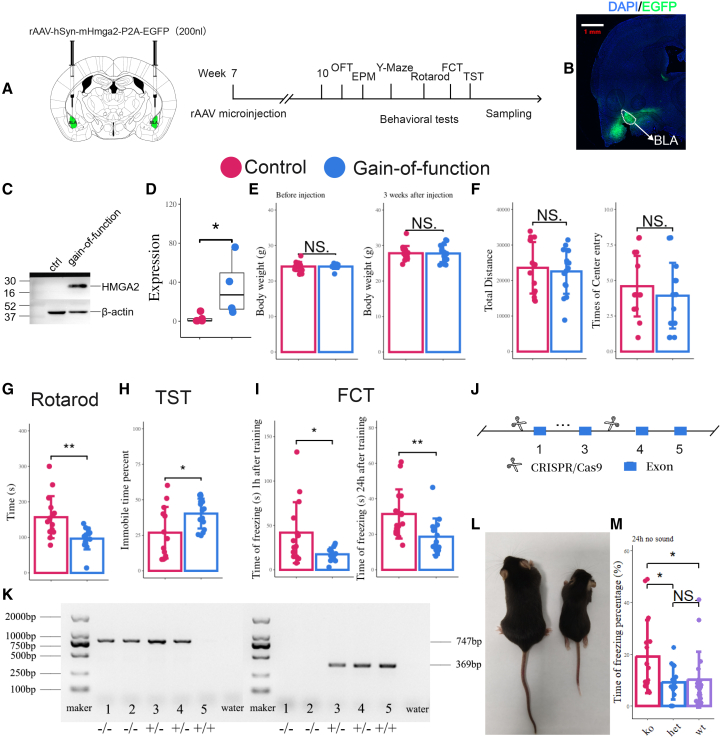
Behavioral experiments using murine models (A) Schematic diagram of basal lateral amygdala virus microinjection and experiment timeline. (B) Expression of EGFP in basal lateral amygdala after virus injection. Scale bar represents 1 mm. (C) Protein level of Hmga2 in BLA of mice given different rAAV virus was detected by western blot. Control: mice given rAAV-hSyn-EGFP as control group. Gain-of-function: mice given rAAV-hSyn-mHmga2-P2A-EGFP, *Hmga2* gain-of-function group. (D) Normalized expression level of *Hmga2* gene derived from RNA-seq data. Control: mice given rAAV-hSyn-EGFP as control group. Gain-of-function: mice given rAAV-hSyn-mHmga2-P2A-EGFP, *Hmga2* gain-of-function group. (E) Histogram plots show body weight of mice at the time of virus injection and 3 weeks after virus injection. NS refers to not significant. *n* = 15 in each group. (F) OFT. Histogram plots show total distance traveled, and times entering into center region of *Hmga2* gain-of-function mice and control mice. (G) Time from start to falling in rotarod test. (H) Tail suspension test (TST). (I) FCT. Significant difference found in time of freezing in FCT 1 and 24 h after training. Gain-of-function group, *n* = 14. Control group, *n* = 15. (J) Mouse *Hmga2* gene KO strategy. Exons 1–3 were deleted. (K) Genotyping strategy using two pairs of primers. (L) Body size of mice at 18 weeks of age. The mouse is wild type on the left and *Hmga2*^−/−^ on the right. (M) *Hmga2*^−/−^ mice have significantly more time of freezing in FCT 24 h after training. ko group: *Hmga2*^−/−^ mice, *n* = 16. het group: heterozygous mice, *n* = 16. wt group: wild-type mice, *n* = 17. Wilcoxon test, ∗*p* < 0.05; ∗∗*p* < 0.01. Data are represented as mean ± SD.

All the four lead SNPs were located in the 3^rd^ intron of the *HMGA2* gene, suggesting the role of this gene in fear (supplemental 2, Table S1). To validate this, we used murine models. This translational approach is supported by the high conservation of gene function across mammals. Moreover, direct invasive functional studies in dogs are ethically and technically challenging, the mouse model thus serves as a rigorous and experimentally tractable system to establish causality. Microinjection of recombinant adeno-associated virus 9 (rAAV9) into specific brain regions enables targeted modulation of gene expression, thereby facilitating functional investigation of genes in behavioral contexts. The amygdala is well-established as a key structure in emotional processing, particularly fear and anxiety, across mammals.^15^ To elucidate the role of *HMGA2* in fear responses, we selectively manipulated its expression in the amygdala. Specifically, gain-of-function of HMGA2 mice was generated via bilateral stereotactic microinjection of rAAV-hSyn-mHmga2-P2A-EGFP into the BLA of male C57BL/6J mice (Figure 2A). The control group was given the same amount of rAAV-hSyn-EGFP bilaterally. Expression of enhanced green fluorescent protein (EGFP) indicates valid injection of rAAV virus into BLA (Figure 2B), but also certain spread to adjacent brain areas. Western blot experiment revealed high expression of Hmga2 protein in the BLA of gain-of-function mice, but almost no expression in the BLA of control mice (Figure 2C), indicating successful expression of *Hmga2* in the BLA. RNA-seq also indicated significantly higher expression of *Hmga2* gene in the gain-of-function mice (Figure 2D). Three weeks later (10 weeks old), a series of behavior experiments were performed to test the effect of *Hmga2* gene on anxiety level, learning, motor ability, fear memory, and depression-like behavior (Figure 2A). Previous studies reported huge effect of *Hmga2* gene on body weight where *Hmga2* KO mice have approximately 60% decrease in body weight.^16^ This massive difference in body size can bias interpretation of behavior test results. We thus recorded mouse body weight pre-injection and 3 weeks post-injection. Body weight did not differ between gain-of-function and control group (Figure 2E), indicating that our virus-based overexpression of *Hmga2* does not influence body weight of mice and can avoid the interference of body size on behavior test.

Neither the open field test (OFT) (Figure 2F), nor the elevated plus maze (EPM) test (supplemental 1, Figure S3A) suggests a difference in anxiety level between gain-of-function mice and control mice. We measured plasma level of a few hormones related to stress, including adrenocorticotropic hormone (ACTH), corticotropin-releasing hormone (CRH), cortisol, and proopiomelanocortin (POMC), using enzyme-linked immunosorbent assay (ELISA) technique. Gain-of-function mice showed no significant difference from controls (supplemental 2, Table S2), suggesting no difference in baseline stress level. Spatial working memory has no implication of significant change in Y maze test (supplemental 1, Figure S3B). Locomotion performance decreased in gain-of-function mice compared to control group (Figure 2G). Meanwhile, gain-of-function mice show increased depression-like behavior in tail suspension test (Figure 2H). In fear conditioning test (FCT) experiments, one gain-of-function mouse died in its home cage after the training phase. Total freezing time significantly decreases in the group of gain-of-function mice in comparison to control group in FCT 1 h and 24 h after training (Figure 2I), indicating decreased contextual and cued fear in gain-of-function mice, independent of body weight.

### Hmga2 KO mice show increased fear memory

We also purchased *Hmga2*-KO mice from GemPharmatech Co., Ltd. Exons 1–3 were deleted in those mice (Figure 2J). These mice serve as loss-of-function models. Genotype of mice was identified according to product of PCR using two pairs of primers (Figure 2K). *HMGA2*^−/−^ mice have much smaller body size relative to wild-type mice, consistent with previous reports.^16^ Despite their smaller body size (Figure 2L), *HMGA2*^−/−^ mice exhibited unexpectedly greater motor capacity (supplemental 1, Figure S3C). Given this substantial size difference, many experimental outcomes are potentially confounded and difficult to interpret directly. Nevertheless, in the FCT experiment, *HMGA2*^−/−^ mice displayed significantly increased freezing time compared to wild-type controls (*p* value 0.012, Wilcoxon test) (Figure 2M). This finding is in line with results obtained from above mice of viral overexpression of *Hmga2* gene.

### HMGA2 regulates embryonic CNS development and postnatal neurotransmitter, synapse, and neurogenesis related pathways

**Figure 3:**
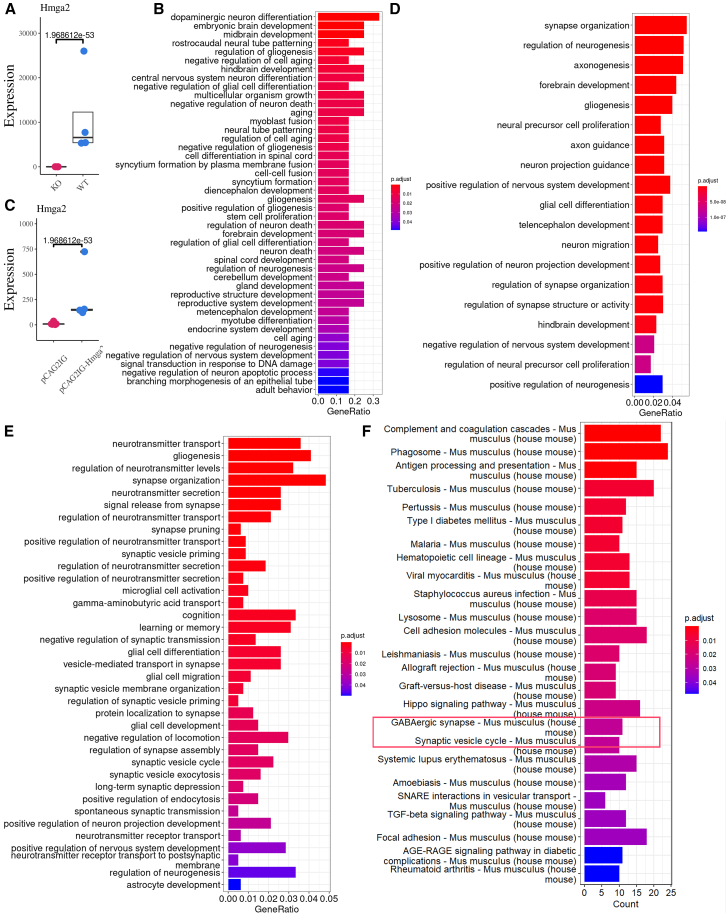
Transcriptomic analyses of three different mouse models (A) Normalized expression level of *Hmga2* gene derived from RNA-seq data. KO: *Hmga2* KO mice, WT: wild-type mice. (B) Enriched BPs by DEGs identified between *Hmga2* KO and wild-type mice. KO: *Hmga2* KO mice, WT: wild-type mice. (C) Normalized expression level of *Hmga2* gene derived from RNA-seq data. pCAG2IG: mice given plasmid without *Hmga2* sequence. pCAG2IG-*Hmga2*: mice given plasmid carrying *Hmga2* coding sequence. (D) Enriched BPs by DEGs from mouse embryonic neocortex with *Hmga2* overexpression. RNA-seq data were obtained from Kuwayama et al.^17^ (E) A subset of CNS development and function-related BPs enriched by DEGs identified between viral overexpression mice and control mice. (F) KEGG pathways enriched by DEGs identified between viral overexpression mice and control mice. The gamma-aminobutyric acid (GABA) transport pathway is enriched.

To understand the mechanism behind the altered fear memory due to *Hmga2* expression change, we obtained RNA-seq from brain of embryos at 12.5 days of gestation, of which three were *HMGA2*^−/−^ and four were wild type. This approach aimed to investigate if *HMGA2* gene influences signaling pathways of neuron development. The rationale for performing RNA-seq on embryonic brain tissues, specifically at the E12.5 stage, is based on the distinct spatiotemporal expression profile of *HMGA2*, where it is highly expressed during early organogenesis and neurogenesis, while be silenced in most adult tissues. Expression of *HMGA2* in KO mice has almost no transcription of *Hmga2* gene (Figure 3A). Transcriptomic analysis revealed 22 differentially expressed genes (DEGs; supplemental 2, Table S3), and these genes are enriched in many biological processes (BPs) related to CNS development and neuron differentiation (Figure 3B).

Kuwayama et al.^17^ overexpressed *HMGA2* gene in mouse embryonic neocortex (C57BL/6J strain) via *in utero* electroporation and obtained RNA-seq data. *HMGA2* gene expression is significantly upper regulated in embryo given pCAG2IG-hmga2 plasmid (Figure 3C). We re-analyzed the data and revealed enriched BPs related to synapse, neurogenesis, and brain development (Figure 3D), also demonstrating role of *HMGA2* gene in CNS development and function. This dataset represents another approach to change expression of the *HMGA2* gene, then identify the genes and signaling pathways regulated by this gene.

We also obtained and analyzed transcriptomes from the amygdala of 15-week-old mice following viral intervention, comparing animals receiving *Hmga2*-overexpressing virus (*n* = 5) with control virus (*n* = 5). One sample of *Hmga2*-overexpression group was excluded from differential gene expression analysis because of undetectable *Hmga2* transcription, probably due to mis-conduction during microinjection operation such as microinjection into wrong site. Consequently, the final analysis included amygdala RNA-seq data from four *Hmga2*-overexpressing mice and five controls. The remaining four amygdala of *Hmga2*-overexpression mice had higher *Hmga2* transcription levels relative to the control group (*p-adjust* 0.01) (Figure 2D). In total, 305 significantly downregulated genes and 550 significantly upregulated genes were identified with a threshold of 0.05 of *p-adjust* value.

Gene ontology (GO) enrichment analysis identified 688 significantly enriched BPs (supplemental 2, Table S4). Majority of these enriched BPs are related to immune responses, which might be explained by the surgery and injection procedures performed. Meanwhile, a variety of BPs related to CNS development and function were identified (Figure 3E). For instance, neurotransmitter-related BPs were enriched, such as neurotransmitter transport (*p-adjust* 3.5E−06), regulation of neurotransmitter levels (*p-adjust* 0.00012), and neurotransmitter secretion (*p-adjust* 0.00020). Synapse-related BPs are also enriched, like synapse organization (*p-adjust* 0.00019), signal release from synapse (*p-adjust* 0.00020), synaptic vesicle exocytosis (*p-adjust* 0.0012), etc. Meanwhile, synaptic compartment enrichment analysis with synGO^18^ also revealed that DEGs are enriched in presynaptic and postsynaptic cellular components (supplemental 2, Table S5. Meanwhile, cognition and learning or memory BPs were enriched (*p-adjust* 0.00016). Besides, BPs related to neuron growth or differentiation were also enriched, such as gliogenesis (*p-adjust* 3.3E−05), regulation of neurogenesis (*p-adjust* 0.00096), positive regulation of nervous system development (*p-adjust* 0.026), glial cell differentiation (*p-adjust* 0.0068), and astrocyte development (*p-adjust* 0.047). Enrichment of these BPs reveals that *HMGA2* can regulate nervous system development and function, thus influencing amygdala-dependent fear.

Kyoto Encyclopedia of Genes and Genomes (KEGG) enrichment analysis was also performed. Most enriched KEGG pathways are related to immune response (Figure 3F), but GABAergic synapse pathway was identified. GO enrichment also identified gamma-aminobutyric acid transport BP (*p-adjust* 0.00011). Many genes specific to GABAergic synapse function were downregulated or upregulated (supplemental 2, Table S6). For instance, *Slc6a13* was upregulated with a fold change of 1.11, which encodes GABA transporter 2 (GAT2) mediating sodium- and chloride-dependent transport of gamma-aminobutyric acid.^19^ *Slc32a1* was downregulated with a fold change of −0.67, which encodes an integral membrane protein involved in gamma-aminobutyric acid and glycine uptake into synaptic vesicles.^20^

### Mutation chr10:8732444T/C locates in a conserved enhancer between humans and dogs

**Figure 4:**
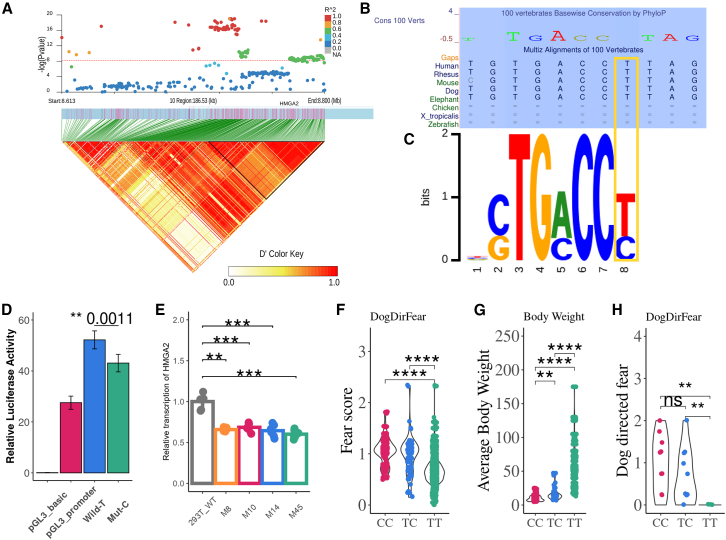
The canFam4 chr10:8732444T/C locates in a putative enhancer and associates with dog-directed fear in Shiba Inus (A) GWAS *p* value and LD with the top SNP, canFam4 chr10:8732444T/C. (B) Basewise conservation of loci flanking canFam4 chr10:8732444T/C (highlighted in the yellow square) estimated by PhyloP from UCSC genome browser. (C) A motif of binding sequence of ESR1 from the Factorbook web repository.^21^ (D) Result of the dual-luciferase reporter gene assay testing the regulatory activities of the mutation in MDCK cell line. pGL3_basic refers to basic plasmid, pGL3_promoter refers to a basic plasmid with promoter; Wild-T and Mut-C refers to pGL3_promoter plasmid carrying either T or C allele, plus flanking 360 bp sequence. (E) Relative mRNA level of *HMGA2* gene in different cell monoclonal derivatives of the HEK293T parental line after genome editing. M45 is the cell clone with heterozygous T>C mutation at the homologous site (hg38 chr12:65853604). M10, M14, and M8 cell clones have an extra InDel besides the heterozygous T>C mutation. All these mutated cells have significantly lower transcription of *HMGA2* gene. (F) Score of DogDirFear of dogs with different genotype. (G) Average body weight of dogs with different genotypes. Dog body weight was represented by the standard breed-average weight. (H) Dog-directed fear level between Shiba Inu dogs of TT (*n* = 5), TC (*n* = 11), and CC (*n* = 10) genotypes. Welch two-sample *t* test, ∗∗*p* < 0.01; ∗∗∗*p* < 0.001; ∗∗∗∗*p* < 0.0001. Data are represented as mean ± SD.

Among the four lead SNPs within significant signals of GWAS results regarding the four fear behavioral traits, canFam4 chr10:8732444T/C emerged as the most significant, with the smallest *p value* (8.72E−20) (Figure 4A; supplemental 2; Table S1). The alternative allele C has a frequency of 0.169 among all 1,987 dog samples sequenced in Dog10K project. Notably, this mutation was not detected either in gray wolves or coyotes within the Dog10K project. The other three most significant SNPs have a frequency of more than 0.1 in 63 Gy wolves included in the Dog10K project (supplemental 2, Table S1).

Due to limited functional annotation of regulatory elements in canFam4 reference genome, we examined the homologous site in human reference genome. Only chr10:8732444T/C homologous site, hg38 chr12:65853604, falls within a known functional element, an enhancer (EH38E3024602). Loci flanking this locus are highly conserved across species (Figure 4B). Intriguingly, no mutation was reported at this homologous locus in humans in gnomAD dataset (v4.1.0) or Common dbSNP (v155), which implies severe consequence of this mutation to health. The other three significant variants either have no homologous site, or no functional elements annotated. Moreover, it is predicted that transcription factor ESR1 binds to the homologous site of chr10:8732444T/C according to SCREEN V3 (Figure 4C).^22^ The human ESR1 is a hormone-responsive nuclear receptor (NR) involved in physiological processes such as cell growth, survival, and cancer metastasis. Activated by its cognate hormone estradiol, ESR1 functions as a homodimer and regulates transcription by binding specific DNA sequences in target genes.^23^

Collectively, these lines of evidence, including strong GWAS association, absence in wild canids, evolutionary conservation, location within a regulatory element, predicted transcription factor binding, and absence in human variation databases, strongly suggest that chr10:8732444T/C is a compelling candidate variant for the observed fear-related behavior in dogs.

To verify the enhancer element in this region of the canine genome and to explore the impact of the mutation on the activity of the enhancer, we performed a dual-luciferase assay in the Madin-Darby Canine Kidney (MDCK) cells. To investigate a candidate enhancer identified in the dog genome, it is crucial to use a cell line with a canine genetic and epigenetic background. MDCK cells were selected as they represent one of the most robust, well-characterized, and commercially available canine cell models with a mature system for genetic manipulation. While a canine neuronal cell line would be ideal, such models are currently not established or commercially accessible. It turned out that the 360 bp sequence flanking the mutation possesses an enhancer activity based on the evidence of considerably higher relative luciferase activity of plasmid carrying flanking sequence relative to an empty plasmid with only promoter sequence (Figure 4D), demonstrating a conserved enhancer element between humans and dogs. Meanwhile, replacing T with C significantly reduces the enhancer activity demonstrated by decreased relative luciferase activity (Figure 4D). The experiment was repeated three times and similar results were obtained. It is therefore expected that allele C would reduce transcription of the target gene via reducing activity of the enhancer. However, it is necessary to investigate in the future whether that the enhancer is active in neuron cells and the mutation alter the enhancer activity.

### The homologous mutation regulates expression of the HMGA2 gene in HEK293T cells

The *HMGA2* gene is likely the target gene of the enhancer given that the mutation is located in the 3^rd^ intron of *HMGA2* gene. Because the locus and enhancer element is highly conserved across species, it is expected that a homologous mutation in human cells would yield similar functional consequence analogous to that in dogs. Currently, gene-editing in dog cell lines is still difficult, and precisely editing neuronal cells remains a significant technical challenge. HEK293T cells provide a highly tractable and mature system for achieving the high-fidelity genome editing required for this study. Meanwhile, *HMGA2* is expressed in the HEK293T cells according to the Human Protein Atlas. To verify whether the specific point mutation within the candidate enhancer directly modulates the expression of its putative target gene, *HMGA2*, we created point mutation HEK293T cell line using Prime editor 3 (PE3) technology. We obtained four different mutated lines (M45, M10, M14, and M8) of HEK293T cells, of which one (M45) is heterozygote for the homologous mutation, the other three have an extra InDel, besides the homologous mutation (supplemental 1, Figure S4). According to quantitative PCR experiment (Figure 4E), transcription of the *HMGA2* gene in mutated cells decreased relative to wild-type HEK293T cells, implicating that T>C mutation at this locus results in lower expression of the *HMGA2* gene. This regulation is likely conserved between humans and dogs, as evidenced by decreased enhancer activity due to the mutation in dogs. Together with results of dual luciferase assay above, chr10:8732444T/C regulates expression of *HMGA2* gene via regulating activity of the enhancer. Meanwhile, mutated cells have lower expression of genes related to cell proliferation, for instance *CDK2*, according to quantitative PCR (supplemental 1, Figure S5). However, if this enhancer function in neuron cells and the mutation influence the expression of HMGA2 in neuron cells, it needs to be investigated further using human neuron cell lines.

### Mutation chr10:8732444T/C associates with fear in Shiba Inu dogs

Dogs with TT (canFam4 chr10:8732444) genotype have significantly lower breed-average fear than dogs of the other two genotypes (TC and CC) (Figure 4F). Difference in fear level between dogs of TC and CC genotype is not significant (Welch’s two-sample *t* test, *p* value 0.07). Genotype frequency in each breed included in Dog10K project is shown in Table S7. Body weight, represented by the standard breed-average weight from American Kennel Club database, is significantly different between different group of genotypes (Figure 4G). There were three Shiba Inu dogs sequenced in Dog10K project. All these three Shiba Inu dogs sequenced in the Dog10K project were all heterozygous at the locus, suggesting genetic diversity within the breed. We sought to identify individuals with homozygous (TT) and heterozygous (TC) genotypes. We first collected buccal swab samples from all adult Shiba Inu dogs (*n* = 36) at a local breeding kennel. All these dogs were kept for breeding purpose under the breeder’s personal selection criteria, which emphasized medium body size and a relatively quiet temperament. Surprisingly, every dog sampled was heterozygous, according to Sanger sequencing (Figure S6). We therefore collaborated with the breeder to genotype several newborn puppies, ultimately obtaining 12 dogs for analysis: 6 homozygous (TT) and 6 heterozygous (TC). All dogs were raised in the same kennel and therefore received the same food, water, care, and other environmental conditions. Using the C-BARQ questionnaire (section 3) completed by the breeder at age ranging from 15 to 28 months, we calculated scores for DogDirFear, StrDirFear, NonSocFear, and TouchSen (Table S8). However, one TT genotype dog was occasionally pregnant and her scores are clear outliers within the TT group (dog TT-1 in Table S8). This dog was therefore excluded from the statistics comparisons. In addition, in collaboration with another breeding kennel, we collected 22 additional Shiba Inu dogs (Table S8). Of these 22 dogs, seven were excluded from subsequent association analysis due to incomplete C-BARQ questionnaires, as certain behavioral scenarios were not observed by the owners and therefore could not be scored. Unfortunately, only one TT dog was identified in this batch of Shiba Inus, and it was removed from association analysis due to incomplete questionnaire. After combining newly collected and previously acquired samples, the final Shiba Inu cohort comprised 26 dogs (15 newly collected +11 from the original cohort), with genotype distribution as follows: 5 TT, 10 CC, and 11 TC.

Analysis of this expanded cohort revealed that TT dogs exhibited significantly lower DogDirFear compared to both TC and CC dogs (Figure 4H). Additionally, TT dogs showed significantly lower StrDirFear than CC dogs (*p* value = 0.03). No significant differences were observed among genotypes for non-social fear or touch sensitivity.

## Discussion

*HMGA2* encodes high-mobility group AT-hook protein 2, which is an abundant non-histone chromatin factor binding to chromosomes and thus regulating chromatin conformation by bending, elongating, curling, coiling, or unraveling it.^24^ Previous studies implied potential association between dog fear (TouchSen) or boldness and *HMGA2* gene, but the association was not validated by behavior experiments.^9^^,^^10^ The present study validated the regulation role of *HMGA2* gene in fear memory via FCT test using mice with virus-based expression of *HMGA2* in BLAs and *Hmga2* KO mice. The behavioral phenotypes observed in our murine models are highly consistent with the functional implications of our GWAS and dual-luciferase assays. Specifically, the T > C mutation, which reduces *HMGA2* transcriptional activity and associates with heightened fear in dogs, mirrors the phenotype observed in *Hmga2*-KO mice. Consequently, while the loss-of-function (KO) model resulted in increased fear levels, the gain-of-function (upregulation) in the BLA led to a significant attenuation of fear. Collectively, these convergent lines of evidence across species and modalities firmly establish a regulatory role for the *Hmga2* gene in fear processing.

### HMGA2 is likely a gene for fear-related mental disorders

**Figure 5:**
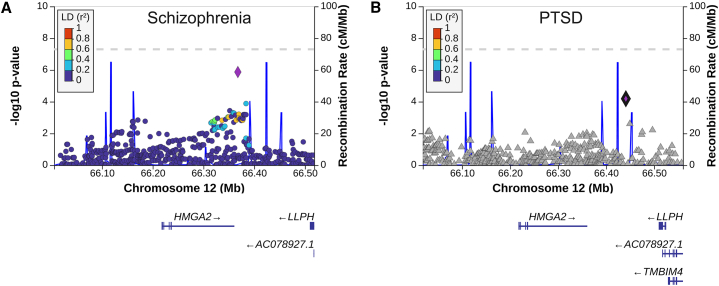
Association between *HMGA2* gene and psychiatric disorders in humans Association between variants in or near *HMGA2* gene and schizophrenia (A) and post-traumatic stress disorder (B) in humans. Data were obtained from studies of Vassily et al. and Nievergelt et al.^36^^,^^37^

Role of *HMGA2* in nervous system development has been recognized in previous studies. In the CNS, *HMGA2* promotes proliferation of neural progenitor cells and confers neurogenic potential on these cells.^25^^,^^26^ Role of *HMGA2* in cell proliferation was also implicated in our mutated cells. Neural stem cells (NSCs) proliferation and self-renew also need *Hmga2* in mice.^26^^,^^27^ Binding of HMGA2 protein to the gene body of highly expressed and neuronal genes is associated with gene repression in embryonic neocortical NPCs.^17^ Meanwhile, *HMGA2* has been associated with various brain development phenotypes in humans in previous studies. An intronic SNP near the 3′UTR of the *HMGA2* gene has the strongest association with intracranial volume in humans in a large scale GWA study.^28^ Locus near *HMGA2* has been previously identified in GWAS on cranial vault shape,^29^^,^^30^ facial morphology,^31^ brain volume,^32^ infant head circumference,^30^ cortical surface area, anteromedial temporal area, posterolateral temporal area, and pars opercularis area,^33^ Moreover, *HMGA2* has also been identified to be associated with insomnia,^34^ subjective well-being,^35^ and schizophrenia^36^ in humans. A variant near *HMGA2* gene is associated with schizophrenia with a *p* value of 1.35E−06 (Figure 5A).^36^ Meanwhile, another variant near *HMGA2* gene is associated with PTSD with a *p* value of 6.50E−05, close to the suggestive threshold (Figure 5B).^37^ The 12q14 microdeletion expanding *HMGA2* gene can cause mild intellectual disability and speech delay.^38^ Association between variants in the 20 kb flanking region and intelligence and late-onset Alzheimer disease in humans has been identified. Taken together, these indicate important roles for *HMGA2* in the development and function of the brain, and imply its potential role in neuropsychiatric diseases in humans. Interestingly, the mutation at a homologous locus has not been reported in humans yet, which implies that a homologous mutation in humans might lead to severe consequences via *cis*-regulatory activity change. In human GTEx dataset, association between *HMGA2* expression and variants in region of 20 kb flanking homologous mutation has not been reported. At the moment of manuscript writing, GTEx project only published data from adult samples. While *HMGA2* is mainly expressed during embryo development, rarely detected after birth in various tissues.^39^ Embryo tissues are needed to identify *cis*-regulatory variants associated with *HMGA2* gene expression level.

Specifically, KEGG enrichment analysis revealed that DEGs are enriched in the GABAergic inhibitory synapse pathway (Figure 3F). It is well known that GABAergic inhibitory control is crucial for the precise regulation of consolidation, expression, and extinction of fear conditioning.^40^^,^^41^ The key role of inhibitory neurotransmission in anxiety disorder has also been universally acknowledged.^42^ Abnormal regulation in GABAergic synapse pathway is an important cause of schizophrenia.^43^ GABA neurotransmitter has also long been known to cause generalized anxiety disorder by dysregulating amygdala activity.^44^ This inhibitory synapse is therefore an important target for medication development for a variety of mental disorders, including schizophrenia and anxiety disorders. For instance, benzodiazepine, a GABA receptor agonist, is widely used in the treatment of a variety mental disorder.^45^^,^^46^

Fear conditioning test has been widely used for studying the etiology and psychopathology of anxiety disorders and PTSD.^47^^,^^48^^,^^49^ Schizophrenia patients also have abnormal conditioned fear.^2^^,^^50^ The association between *HMGA2* and both social and non-social fears in dogs identified in the current study, in addition to reduced fear of mice due to *Hmga2* overexpression revealed by the FCT test, and its regulation role in inhibitory synapses which is related to human fear- or anxiety-related disorders, collectively reveal the regulatory role of *HMGA2* in fear and suggest that *HMGA2* is very likely a novel candidate gene for fear-related mental disorders. Dogs with high fear due to *HMGA2* gene risk mutations could be a good model for fear-related mental disorder study.

### A pleiotropic gene co-regulating morphological-behavioral traits

The convergent role of *HMGA2* in regulating body size/height has been identified across a variety of species. A series of *Hmga2* KO mice were created with various strategies. All of those KO mice manifested dwarfism trait.^16^^,^^51^^,^^52^ Additionally, body size regulation in a dosage-dependent manner was observed in rabbits^53^ and pigs.^54^ A nonsynonymous mutation in *HMGA2* gene has been associated with smaller stature in Shetland ponies.^55^ In dogs, GWAS analyses also revealed strong association between *HMGA2* gene and body size.^56^^,^^57^ Moreover, common variants in *HMGA2* gene have been associated with human height.^58^ *HMGA2* variants have been identified to be causes of Silver-Russell syndrome (SRS), a heterogeneous disorder characterized by intrauterine and postnatal growth retardation.^59^ This gene also strongly correlates with beak size in Darwin’s finches.^60^

Besides role in body size, *HMGA2* has been an important subject in the onset of cancer, cell cycle, and the apoptosis process.^61^ Meanwhile, the reproductive system is also under regulation of *HMGA2*. *Hmga2*^−/−^ mice are infertile and show abnormal development in reproductive tissues.^16^^,^^54^ *HMGA2* was also found to be highly associated with inguinal cryptorchidism risk in dogs.^62^

Body size correlates with fear behavior. McGreevy et al.^63^ revealed significant correlation between height and fear in a negative way. Domestication experiments demonstrate correlation between fear and a variety of morphological traits, such as piebald markings, altered body mass and increased reproductive capability, known as domestication syndrome.^64^^,^^65^ A negative correlation between fear level and body size in red junglefowl was observed. Jensen et al. continually selected red junglefowl solely on lower fear of human, resulting difference in body size between low-fear line and high-fear line.^66^ However, a positive correlation between fear and body weight has also been reported, for example in mice.^67^ The complexity and heterogeneity of genetics underlying behavior and body size may explain this conflicting correlation. Effect of *HMGA2* on reproduction was not investigated in the present study, however, previous studies reported infertility due to *HMGA2* KO in mice and pigs.^16^^,^^54^

Previous study revealed reduced exploratory behavior by OFT, nest building, and marble burying tests in *Hmga2* KO mice.^16^ This study uncovered regulation of fear memory by *Hmga2* gene using two different mice models. Furthermore, our transcriptome analyses from three different mice models revealed that *Hmga2* in mouse influences the expression of a series of genes involving in BPs related to neurotransmitter, synaptic activities, neurogenesis, and early brain development during gestation.

Role in nervous system development and function, growth, and sexual system development strongly demonstrates that *HMGA2* is a pleiotropic gene co-regulating morphological, behavioral, and reproductive traits, and supports the pleiotropic mechanism hypothesis leading to domestication syndrome. Due to its broad and considerable effect on body size and role in regulating nerve system function and fertility, molecular pathways regulated by *HMGA2* deserve further investigations.

### Limitations of the study

We validated an enhancer using MDCK cell line and reported a mutation in that enhancer that can regulate the expression of the *HMGA2* gene in HEK293T cell line. While this demonstrates the regulatory potential of the variant, it does not confirm its activity in fear-related brain circuits. Future studies using canine neural cell models or brain epigenomic profiling would be valuable to establish tissue-specific enhancer activity. The fear-associated trait under investigation is primarily regulated by the CNS. However, the cells utilized in both the dual-luciferase reporter assays and the gene-editing experiments were non-neuronal. While neuronal cells would constitute a more biologically relevant model, there is currently no established, mature system for employing canine neural cells in such functional assays, and achieving precise genetic editing in neuronal cell lines remains technically challenging. Therefore, future research should prioritize investigating the role of the candidate mutations specifically within neural cells to better elucidate their impact on *HMGA2* regulation.

Furthermore, it is plausible that multiple non-coding regulatory variants form complex haplotypes, collectively leading to quantitative differences in *HMGA2* expression. Although this study functionally validated only the most significantly associated variant, other variants in strong linkage disequilibrium (LD) may also influence *HMGA2* by affecting additional *cis*-regulatory elements. Thus, we cannot exclude the possibility that nearby variants contribute to the regulation of both fear and body size.

In this study, we validated the association between the candidate variant and dog-directed fear within a single breed, the Shiba Inu. However, the sample size is limited, especially samples of TT genotype. Further validation in a larger cohort of a single breed (not only the Shiba Inu, but also other breeds), ideally under consistent living conditions, is required to confirm the association. Such expanded studies could also enable the use of dogs with different genotypes as models to investigate the mechanisms linking genetic variation to fear behavior and body weight regulation.

Finally, while we observed significant associations between the *HMGA2* genotype and both body weight and fear-related scores, it remains unclear whether a single variant is pleiotropically responsible for both phenotypes. It has yet to be determined whether these effects stem from overall *HMGA2* expression levels or if distinct regulatory variants independently modulate each trait, potentially by affecting expression in different cell types through mutations in separate regulatory elements. Notably, the fear-associated locus identified here is distinct from previously reported body size QTLs (e.g., on chr10:8808920). This specific variant also showed a significant association with dog-directed fear (*p* value 2.78E−07, ranking 635th) in our prior GWAS.^56^ To dissect these mechanisms, the generation of point-mutated mouse models, as well as the use of point-mutated various cell lines including neural progenitors, would be highly valuable.

## Resource availability

### Lead contact

Requests for further information and resources should be directed to and will be fulfilled by the lead contact, Ya-Ping Zhang (zhangyp@mail.kiz.ac.cn).

### Materials availability

There are restrictions to the availability of gene edited HEK293T cells and Hmga2 knockout mice because of the lack of an external centralized repository for its distribution and our need to maintain the stock. We are glad to share them with reasonable compensation by requestor for its processing and shipping.

### Data and code availability

• Variant file in vcf format from the Dog10K project was collected from website https://kiddlabshare.med.umich.edu/dog10K/. RNA-seq data have been deposited at Genome Sequence Archive (GSA) as CRA016531 and are publicly available as of the date of publication.
• Original western blot images have been deposited at Mendeley at (https://doi.org/10.17632/b27xm6wcr6.1) and are publicly available as of the date of publication. Microscopy data reported in this paper will be shared by the lead contact upon request.
• This paper does not report original code.
• Any additional information required to reanalyze the data reported in this paper is available from the lead contact upon request. The C-BARQ data reported in this study cannot be deposited in a public repository because of copyright restrictions. C-BARQ behavior data were provided by James Serpell. To request access, contact James A. Serpell for requesting access.

## Acknowledgments

We thank Ming Li from Kunming Institute of Zoology CAS for his suggestions to the study. We thank Zuoren Wang for his suggestions on behavioral experiments of mice. We thank Jianxia Xue from Kunming Institute of Zoology CAS for her work in collecting the dog sample and questionnaire. We also thank James Serpell and the University of Pennsylvania for sharing the C-BARQ data. We thank the Animal Bank at the Germplasm Bank of Wild Species (https://cstr.cn/31121.02.GBOWS.AB) for providing biological materials and technical support. Particularly, we thank those lab mice sacrificed in the study. This work was supported by the STI2030-Major Projects (2021ZD0203900), 10.13039/501100001809National Natural Science Foundation of China (32302737), 10.13039/501100012166National Key R&D Program of China (2019YFA0707101), Spring City Plan: the High-level Talent Promotion and Training Project of Kunming (2022SCP001), Key Research and Development Program of Yunnan province (202203AC100010), Key Research Program of Frontier Sciences of the Chinese Academy of Sciences (CAS) (ZDBS-LY-SM011), Project of Qingdao Emerging Industry Cultivation Plan (23-1-4-xxgg-21-nsh), Shandong Province Small and Medium-Sized High-Tech Enterprise Innovation Capacity Enhancement Project (2023TSGC0506), and Yunnan Fundamental Research Projects (202401CF070061).

## Author contributions

Conceptualization, Y.Y., Y.L., and Y.P.Z.; methodology, Y.Y., C.L., Y.S., X.W., Y.L., S.Z., J.R., and Y.L.; investigation, Y.Y., C.L., Y.S., X.W., Y.L., S.Z., J.R., J.A.S., and Y.L.; visualization, Y.Y.; supervision, Y.Y., J.R., Y.L., and Y.P.Z.; writing – original draft, Y.Y.; writing – review and editing, J.A.S., J.R., Y.L., and Y.P.Z.; funding acquisition, Y.Y. and Y.P.Z.; resources, J.A.S., J.R., and Y.L.; supervision, Y.P.Z.

## Declaration of interests

The authors declare that they have no competing interests.

## STAR★Methods

### Key resources table

**Table:** 

REAGENT or RESOURCE	SOURCE	IDENTIFIER
**Antibodies**		

HMGA2 antibody	Abcam	ab97276; RRID: AB_10679322
β-actin antibody	ABclonal	AC004; RRID: AB_2737399
anti-rabbit	Cell Signaling Technology, Inc.	7074S; RRID: AB_2099233
anti-mouse	Cell signaling	7076S; RRID: AB_330924

**Deposited data**		

Mutation file in vcf format	Dog10K project	https://kiddlabshare.med.umich.edu/dog10K/
RNA-seq data	Genome Sequence Archive	CRA016531

**Experimental models: Cell lines**		

MDCK cell line	Conservation Genetics CAS Kunming Cell Bank	CVCL_0422
HEK293T	American Type Culture Collection	CVCL_1926

**Experimental models: Organisms/strains**		

*Hmga2* knockout mice	GemPharmatech Co., Ltd	T009335

**Oligonucleotides**		

ATTAAGCGAGTCAATGGCTAGTTTTAGAG CTAGAAATAGCAAGTTAAAATAAGGCTAGT CCGTTATCAACTTGAAAAAGTGGCACCGAG TCGGTGCTCTGTGACCCTAGCCATT GACT2CGCTTAAtttttttt	pegRNA	-
CCTAAGTCAGTGATTCTGAG	sgRNA	-
GCCATGAGGACCGTCCAAGA	Forward primer	Dog
GAAGCCTAGCCGCAAACCAG	Reverse primer	Dog

**Software and algorithms**		

Gemma	https://github.com/genetics-statistics/GEMMA	-
STAR	https://github.com/alexdobin/STAR	-
DESeq2	https://bioconductor.org/packages/release/bioc/html/DESeq2.html	-
RSEM	https://github.com/deweylab/RSEM	-
clusterProfiler	https://guangchuangyu.github.io/software/clusterProfiler/	-
Fastp	https://github.com/OpenGene/fastp	-

### Experimental model and study participant details

#### Genotype and phenotype data

The SNP and InDel variants were obtained from the Dog10K project.^14^ Briefly, raw sequencing reads were aligned to a modified version of the UU_Cfam_GSD_1.0 (GCF_011100685.1). Read data was processed across multiple centers using a shared GATK-based pipeline prior to centralized genotyping and filtration of candidate variants on the autosomes and chrX. Following alignment, samples were removed due to low coverage (< 10 ×), the presence of sample duplicates, mislabeled or unknown breed identity, or potential contamination indicated by reference read fraction at heterozygous positions. To identify candidate SNVs, we applied the Variant QualityScore Recalibration (VQSR) procedure with cutoffs that retain 99% of variants present onthe Illumina CanineHD BeadChip and Axiom K9 HD genotyping arrays. Quality control was applied before further analyses based on mutation data using plink under criteria as followings. First, only biallelic SNPs and InDels were kept. Variants with minor allele frequence less than 0.05 were removed, variants with a miss genotyping rate of more than 0.1, and individuals with missing calling ratio greater than 0.1, were also removed. For the 4 kinds of fear surveyed in C-BARQ, an average score for each breed was calculated.

#### Generation and genotyping of *Hmga2* knockout mice

The *Hmga2* knockout mice (C57BL/6J) were generated by GemPharmatech Co., Ltd. (Nanjing, China), with the targeted deletion encompassing exons 1–3 of the Hmga2-203 transcript (ENSMUST00000159699.1). Founder mice carrying heterozygous Hmga2 mutations (two males and two females) were housed in a specific pathogen–free (SPF) facility and bred in pairs. Following breeding expansion, three genotypes were obtained: wild-type, heterozygous, and homozygous knockout. Genotyping was performed on pups 5–7 days after birth using tail clipping and toe numbering. Genomic DNA was extracted from tail tissues, amplified by polymerase chain reaction (PCR), and analyzed via polyacrylamide gel electrophoresis (PAGE). The wild-type genotype was identified by a 369 bp band, heterozygotes displayed both ∼747 bp and 369 bp bands, and homozygotes exhibited only the ∼747 bp band.

### Method details

#### Genome wide association analysis

Gemma^68^ was used to perform the genome-wide association analysis using linear mixed models. Average score of each behavior was used as the phenotype of the breed in GWAS analysis. Genomic relationship matrix was calculated and used as covariate in the GWAS analysis. Manhattan plot was plotted in R and a Bonferroni corrected threshold was calculated as the threshold of significance.

#### Dual-luciferase assay

Dual-luciferase assays were performed to confirm if the DNA fragments containing either T or C allele at canFam4 chr10:8732444 showed different enhancer activity. The DNA sequence covering canFam4 chr10:8732444 site (including either T or C allele) from chr10:8732264 to chr10:8732624 were cloned into pGL3-Promoter luciferase reporter plasmid. Sanger sequencing was used to verify accurate construction. Then, pGL3-basic, pGL3-Promoter, and the two constructs were separately transfected into Madin-Darby canine kidney (MDCK) cells using jetPRIME® *in vitro* DNA & siRNA transfection reagent (Polyplus, Illkirch, France) in 24-well plates (six wells per each vector, 500ng vector/well). The pRL-TK Renilla Luciferase vector acted as the internal control and was co-transfected into cells with the above luciferase report plasmid (25ng vector/well). The firefly and renilla luciferase activities were measured according to the manufacturer’s instruction (Promega) 24h after transfection. The final firefly luciferase activity was normalized to the renilla luciferase value. This experiment was repeated 3 times independently.

#### Point mutation editing in HEK293T cells

First of all, the PE3 expression vector was constructed, which incorporates a pegRNA specifically designed to target the mutation site, a nick sgRNA, and an nCas9-RT fusion protein. Sequence of pegRNA: ATTAAGCGAGTCAATGGCTAGTTTTAGAGCTAGAAATAGCAAGTTAAAATAAGGCTAGTCCGTTATCAACTTGAAAAAGTGGCACCGAGTCGGTGCTCTGTGACCCTAGCCATTGACTCGCTTAAtttttttt. Sequence of sgRNA:CCTAAGTCAGTGATTCTGAG. Then, 2.5 μg of plasmid DNA was transfected into HEK293T cells in a 6-well plate using the jetPRIME® transfection reagent (Polyplus). Limiting dilution was performed after 5 days of incubation. Ten days later, half of the single-cell clones were used for genotyping. Primer sequences used for PCR are as follow: forward primer: AGAAGCAAGAAACTTCCCCC, reverse primer: TGGAACTGCATTCCCACACA. Sanger sequencing was performed to identify positive clones for further research.

#### Cell culture

The MDCK cell line (RRID: CVCL_0422) was supplied by Conservation Genetics CAS Kunming Cell Bank (Kunming, China). The HEK293T cell line was purchased from the American Type Culture Collection (ATCC), catalog number CRL-11268 (RRID: CVCL_1926). MDCK cells was cultured in Dulbecco’s modified Eagle’s medium (DMEM). The medium was supplemented with 10% fetal bovine serum (FBS) and 1% Penicillin-Streptomycin. There were no additional penicillin and streptomycin to the culture medium during cell transfection. MDCK cells were cultured at 37 °C in an incubator with 5% CO_2_. The HEK293T cells were maintained in growth medium containing 89% DMEM/H, 10% fetal bovine serum (FBS) (Gibco), 100 U/ml penicillin and 100 μg/ml streptomycin (Gibco) at 37°C in a 5% CO2 atmosphere with saturated humidity. Cell lines were confirmed to be free from contamination (including mycoplasma, bacteria, and fungi). We used cells within 10 passages to ensure integrity.

#### Stereotactic microinjection of recombinant adeno-associated virus (rAAV)

Male mice (C57BL/6J) were bilaterally injected with rAAV-hSyn-mHmga2-P2A-EGFP (titer: 5.99E+12) or rAAV-hSyn-EGFP (titer: 5.76E+12) in to BLA at age of 7 weeks, both viruses were provided by Brain Case Biotechnology (Shenzhen, China). The rAAV-hSyn-mHmga2-P2A-EGFP carries coding sequence of mice *Hmga2* gene (NM_010441.3). All animal procedures were performed in strict accordance with the guidelines established by the Institutional Animal Care and Use Committee (IACUC) of the Kunming Institute of Zoology, Chinese Academy of Sciences. The study protocol was formally approved under the permit number IACUC-RE-2023-12-002.

Mice were anesthetized by intraperitoneal injection of Avertin (0.2 ml/10 g according to body weight). The hair on the skull of the mice was removed with scissors and cleaned up to expose the scalp. The head was then fixed on the rat brain stereotaxic apparatus (RWD Life Science Co., Ltd., Shenzhen, China). The tongue was pulled out gently with forceps to prevent mice from suffocating due to poor respiration caused by sputum entrapment during the procedure. After that, local anesthesia was done with subcutaneous injection of 0.2 ml procaine into the cranial surgical area. The tissues on the scalp were clipped then, and cranial bones were wiped gently with cotton swabs moistened with peroxide and the remnants of the tissues were clipped away. The BLA (-1.4 cm, ±3.22 cm, -4.6cm) was then located according to The Mouse Brain in Stereotaxic Coordinates 2^nd^ edition compiled by Paxinos and Franklin.^69^ A 0.5 mm cranial drill (RWD Life Science Co., Ltd., Shenzhen, China) was used to drill through the skull at the marked locations. A sterile cotton tip was used to wipe away any blood from the hole. Then 500 nl of adeno-associated virus solution was aspirated using the micro-syringe (RWD Life Science Co., Ltd., Shenzhen, China), after which the needle was positioned to the surface of the skull to record the height of the needle. Then needle was slowly lowered to the BLA according to the depth of the desired brain region, and 200 nl viral solution was injected at a rate of 2nl/s. After injection, wait about 8 minutes to ensure the virus to be fully absorbed. Then slowly raise the syringe and remove any liquid from the injection site. The wound was sutured carefully and wiped with a sterile cotton tip. Finally, the mice were gently removed from the stereotaxic apparatus and placed in a cage with clean bedding to wait for the mice to wake up and return to their home cages. All protocols were approved by the Institutional Animal Care and Use Committee (IACUC: IACUC-RE-2023-12-002) of the Kunming Institute of Zoology, Chinese Academy of Sciences.

#### Behavior experiments

Open field test, elevated plus maze, Y-maze, Rotarod test, Fear conditioning test, and Tail suspension test were performed sequentially. All behavioral experiments were performed during the day (9:00 a.m. to 6:00 p.m.) in a soundproof room at room temperature. Each type of tests was performed in a relatively fixed time during the day. There were 2- or 3-days interval between different tests. Mice were transferred to test room 1h before test for habituation. Group identity of the tested mice was unknown to the experimenter. The inner surfaces of instruments were cleaned with 75% ethanol after each test.

#### Open field test

The experiment was performed in a 50cm × 50cm × 40cm closed box with a white bottom. The bottom of the box was divided into three zones, namely the center zone, the corner zone, and the edge zone. Mice were placed in the behavioral test room for about 1 h before the experiment to acclimatize to the environment. VisuTrack Behavioral Analysis and Recording Software (Shanghai XinRuan Information Technology Co., Ltd., Shanghai, China) was used to record trajectory of mice for 5 minutes. Before the formal test, a non-experimental mouse was used to test whether the tracking system could work well.

#### Elevated plus maze

The elevated plus maze consists of a pair of open arms (50cm × 10cm, L × W) and a pair of closed arms (50cm × 10cm, L × W) with a height of 50cm from the floor. The mice were put into the central area with head facing the one of open arms. VisuTrack Animal Behavioral Analysis Software was used to record the trajectory of the mice in the apparatus for 5 minutes. Total distance traveled in the maze and the percentage of time in the open arms were calculated.

#### Y-maze

The Y-maze consists of three arms of equal length, namely the novel arm, the start arm and the memory arm. The angle between two adjacent arms is 120°, and there is a door in the center into each arm. Different geometric shapes were affixed inside each arm as visual markers. At the beginning of the experiment, the entry to novel arm was closed and the mouse was given 10 minutes to explore the different arms from start arm. After this, the entry to the novel arm was open and the mouse was gently put back to the start arm. VisuTrack software was used to track the animal's trajectory for 5 minutes. After all these finished, the mouse was put back to home cage.

#### Rotarod-test

Acclimate mouse to the test environment and handler before testing. Bring mouse in their home cage to the behavioral room at least 1 hour before testing to minimize stress. Before the experiment, 2 consecutive days of training were required, 1 time per day. Place mouse in separate lanes on the rod rotating at 10rpm/min, allowing them to walk forward to maintain balance for 10 min. On the second day, place mouse in separate lanes on the rod rotating at 20rpm/min, allowing them to walk forward to maintain balance for 10 min. Place mouse in separate lanes on a rod starting at 4 rpm. Set the Rotarod to accelerate from 4 to 40 rpm over 300 seconds. The trial begins with the start of acceleration and ends when a mouse falls off the rod. If a mouse clings to the rod and completes a full passive rotation, the timer for that mouse is stopped. Return any fallen mouse to their home cage, ensuring minimal disturbance to others still in the trial. A repeat trial is conducted if the mouse passively rotates or falls off within 5 seconds of the trial start.

#### Fear conditioning test

The fear conditioning chamber was equipped with two side walls of striped pattern (Super Fear Conditioning Analysis System, Xinruan, Shanghai, China). This experiment lasts 3 days. During day one, mouse was placed in the chamber for 10 minutes for acclimation to the experimental environment and reduction of orienting responses. On the second day, mouse was placed in the chamber and 2 minutes later it was given the first neutral conditioned stimulus (auditory cue, 18s, 98 db, 2kHz) unconditioned stimulus (US) (electric footshock, 0.7 mA, 2 s), coupled 3 times with a 2-min interval between each coupling. After that the mouse was placed back into home cage. Contextual fear was tested 1 hour after training. Mouse was placed back in the fear conditioning chamber and their contextual freezing behavior was measured for 11 minutes without any foot shocks applied. Cued fear was tested 1 day after training on the third day. Mouse was given auditory cue matching the training period (18 s, 98 db, 2 kHz), with a sound interval of 2 min, and no plantar foot shock. The movement of the mice was recorded using a near-infrared camera and analyzed in real-time with VisuFcs software (Shanghai XinRuan Information Technology Co., Ltd., Shanghai, China).

#### Tail suspension test

The mouse was suspended using a piece of adhesive tape. The tape is strong enough to prevent the mouse from falling and does not cause damage to the skin of the tail. The test lasts for 6 min. VisuTrack software was used to record and analyze the state of the mouse. The percentage of immobility time (defined as the time during which the animal is hanging passively and motionless) is measured for each animal, and considered as an index of “depression-like” behavior.

#### Mouse embryo collection

Mouse embryos were collected from pregnant C57BL/6J female mice on day 12.5 of pregnancy (E12.5). The pregnant females were anesthetized with tribromethanol (Alphadin), and the entire uterus was harvested. The uterus was washed three times with cold PBS. Under a stereomicroscope, the uterus was carefully dissected, and each embryo was identified and numbered. The surface of each embryo was gently wiped with dust-free paper to remove any excess liquid, and the brain of each embryo was isolated. Some tissues were also collected for DNA extraction for subsequent genotype identification. The tissues were placed in cryotubes and transferred to a −80 Celsius degree freezer, snap-frozen and stored.

#### RNA-seq

Mouse was anesthetized using isoflurane and blood was then collected from retro-orbital sinus after removing eyeball one week after all behavior experiments finished. The mouse was decapitated, and the whole brain tissue was taken. Intact BLA tissue was collected according to a brain atlas^69^ and stored in -80 Celsius degree condition. RNA library construction and pair-end sequencing were performed by Novogene (Tianjin, China) using Illumina Novaseq 6000. Quality control on sequencing data was performed using fastp tool.^70^ STAR^71^ was used to map reads to mm10 reference genome. RSEM^72^ was used to count expression of each gene in each sample. DESeq2 package^73^ was used for differentially expressed gene identification. Gene ontology (GO) and Kyoto Encyclopedia of Genes and Genomes (KEGG) enrichment analysis were performed using clusterProfiler package.^74^ Web-based tool synGO was used to perform synaptic compartment enrichment analysis.^18^

#### Real-time quantitative PCR

Total RNA was extracted using TRIzol™ reagent (Invitrogen) and the concentration of RNA was determined by Nano-Drop ND 2000c Spectrophotometer (Thermo Scientific). Then, 500ng RNA was reverse-transcribed to cDNA with HiScript III 1st Strand cDNA Synthesis Kit (+gDNA wiper) (Vazyme). Real-time quantitative PCR was performed by QuantStudio 5 system (Applied Biosystems) using the ChamQ Universal SYBR qPCR Master Mix (Vazyme) in a final volume of 20 μl. Relative expression of mRNA was calculated using 2^-ΔΔCt^ method with GAPDH mRNA as endogenous control. Primer sequences are listed in the Table below.

**Table:** Table. Primer sequences used for Real-time quantitative PCR.

Gene name	Primer	Sequences (5′-3′)	GenBank accession no.	Product size (bp)
human GAPDH	Forward Reverse	TCAAGAAGGTGGTGAAGCA GTCAAAGGTGGAGGAGTGG	NM002046.7	116
human HMGA2	Forward Reverse	GAAGCCACTGGAGAAAAACGGC GGCAGACTCTTGTGAGGATGTC	NM003483.6	114
human CDK1	Forward Reverse	TATGCCTTGGTCAGAGTA ATGGCTGCTAATAAACAC	NM001320918.1	100
human CDK2	Forward Reverse	GCCAACTCTGGGAATACA CCAACCCTCTTCAGCAAT	NM001798.5	131
human PCNA	Forward Reverse	CTGTAGCGGCGTTGTTGC CGTTGATGAGGTCCTTGAGTG	NM002592.2	103

#### ELISA

The blood was centrifuged at 3000g for 30 minutes and then plasma was separated. ELISA was performed to detect plasma level of 5 hormones, namely GH, ACTH, CRH, cortisol, and POMC, using kits of MM-43739M1, MM-0509M1, MM-0554M1, MM-0565M1 (Meimian Industrial Co., Ltd, Jiangsu, China), CSB-EL018363MO (CUSABIO, Wuhan, China) according to manufacturer’s instructions, respectively.

#### Western blot

Western blot analysis was performed to determine the protein levels of HMGA2.^75^ Briefly, proteins of BLA were extracted using RIPA lysis and Extraction Buffer (89900, Thermo Fisher Scientific), complemented with complete protease inhibitor cocktail (78430, Thermo Fisher Scientific). Protein lysates (30μg/lane)were separated on 10% SDS-PAGE gel (E303-01, Vazyme) and transferred onto PVDF membranes (IPFL00005, Merck Millipore), and incubated at 4°C overnight with the primary HMGA2 antibody (1:2000, ab97276, Abcam) or β-actin antibody (1:2000; AC004, ABclonal). The membranes were then washed and incubated with either anti-rabbit (1:2000; 7074S, Cell Signaling Technology, Inc.) or anti-mouse (1:2000; 7076S, Cell signaling) secondary antibodies conjugated to HRP. The bands were visualized by Clarity™ Western ECL Substrate (1705060, BIO-RAD) on the Gel Imaging System (Tanon).

#### Genotyping of Shiba Inu

Oral epithelial cells were collected from neonatal Shiba Inu using Whatman buccal swabs by the owner and sent to us by post. Genomic DNA was extracted using the Buccal Swab Genomic DNA Extraction Kit (Cat. No. EE201-01) from Beijing TransGen Biotech Co., Ltd. (Beijing, China). The concentration and purity of the extracted DNA were quantified using a NanoDrop 2000 spectrophotometer (Thermo Scientific, USA). Primers were designed with Primer Premier 5.0 software based on the reference sequence from GenBank (Accession No. 100271859). The sequences of the primers were as follows: forward primer (F): GCCATGAGGACCGTCCAAGA, reverse primer (R): GAAGCCTAGCCGCAAACCAG, yielding a 700 bp amplification product. The target sequence was amplified via polymerase chain reaction (PCR), and the resulting products were analyzed by polyacrylamide gel electrophoresis (PAGE). After verifying the amplification, Sanger sequencing (from Shanghai Sangon Biotech) was performed, and the obtained sequences were aligned with the reference sequence. Sequence alignment and variant annotation were conducted using SnapGene software for visualization.

#### Statistical analysis

Data are presented as means ± s.d. and were compared between groups with two-tailed Student’s t test, Welch Two Sample t-test, or Wilcoxon test using R tool. Chi-square test was performed using R tool as well.

### Quantification and statistical analysis

Please describe here all statistical analysis and software used. We ask authors to indicate in this section where all of the statistical details of experiments can be found (e.g., in the figure legends, figures, results, etc.), including the statistical tests used, exact value of n, what n represents (e.g., number of animals, number of cells, etc.), definition of center, and dispersion and precision measures (e.g., mean, median, SD, SEM, confidence intervals). Also, please summarize in this section how significance was defined, the statistical methods used to determine strategies for randomization and/or stratification, sample size estimation, and inclusion and exclusion of any data or subjects, as well as any methods used to determine whether the data met assumptions of the statistical approach.

## Supplemental information

Document S1. Figures S1–S6

Table S1. The most significant variant identified for four fear traits

Table S2. ELISA results

Table S3. Differentially expressed genes identified from embryos of HMGA2 knockout mice

Table S4. Enriched biological process by differentially expressed genes

Table S5. Synaptic compartment enrichment analysis of differentially expressed genes (DEGs) with synGO

Table S6. Differentially expressed genes involving in GABAergic synapse function

Table S7. Counts of dogs of different genotype in each breed of Dog10K project

Table S8. Results of questions in section 3 of C-BARQ in Shiba Inu dogs of different genotypes
