# Supplementary material for: HMGA2 regulates fear and growth: Canine GWAS and functional evidence
Source: iScience. 2026 Jun 17;29(7):116429. doi: 10.1016/j.isci.2026.116429 (PMC13293657; doi:10.1016/j.isci.2026.116429)
Supplement: Document S1. Figures S1–S6 [file mmc1.pdf]

iScience, Volume *29*

Supplemental information

HMGA2 regulates fear and growth: Canine GWAS and functional evidence

Yun Yu, Chao Li, Ye Liu, Yinyu Su, Xuebin Wang, James A. Serpell, Shurun Zhang, Jinxue Ruan, Yanhu Liu, and Ya-Ping Zhang

Supplementary materials

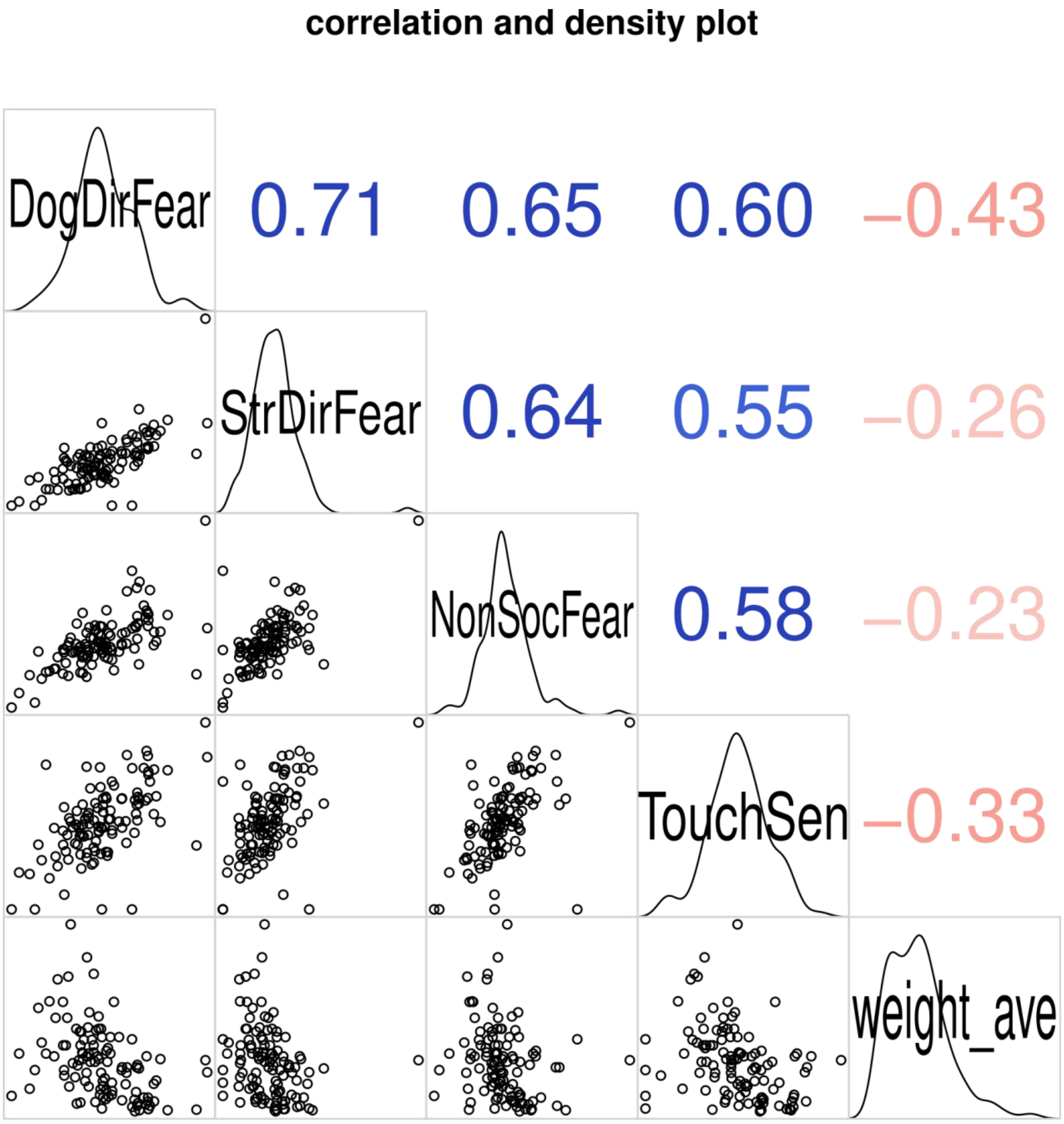

Figure S1. Correlation test between breed-average weight (weight_ave), dog directed fear (DogDirFear), stranger directed fear (StrDirFear), non-social fear (NonSocFear), and touch sensitivity (TouchSen), related to Figure 1. Density distributions of these 5 traits were presents on diagonal panel. Upper panel shows pairwise Pearson's correlation coefficient and lower panel shows pairwise dotplot.

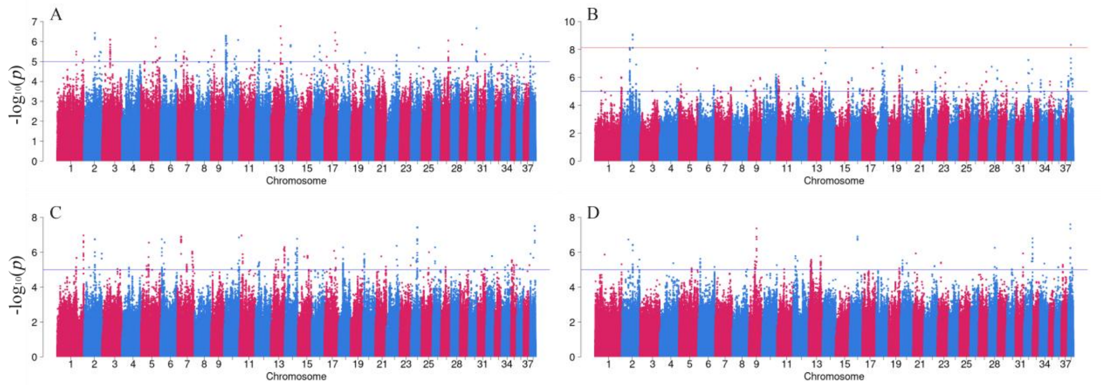

Figure S2. Manhattan plots of the GWAS for fear behavior, adjusting for breed-average body weight, related to Figure 1. (A-D) Manhattan plots showing the association of genome-wide variants with DogDirFear, referring to unfamiliar dog directed fear (A), StrDirFear, referring to unfamiliar person directed fear (B), NonSocFear, referring to non-social fear (C), TouchSen, referring to touch sensitivity (D) after including breed average body weight as covariate.

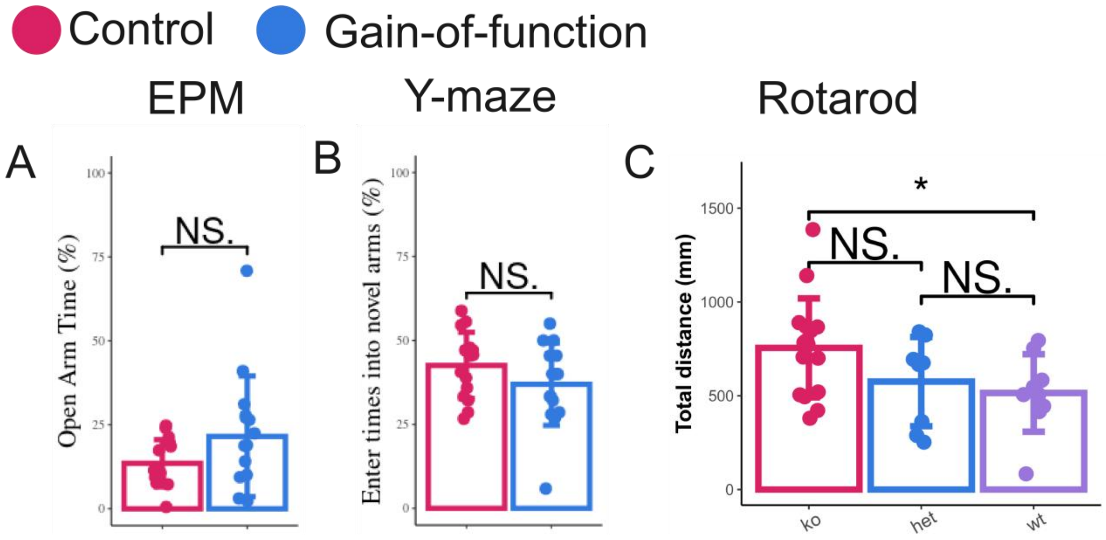

Figure S3. Results of behavioral tests of mice, related to Figure 2. (A) Histogram plots show time spent in the open arm of elevated-plus maze. n=15 in each group. (B) Percentage of times entering into novel arms. n=15 in each group. Gain-of-function group was injected with rAAV-hSyn-mHmga2-P2A-EGFP. Control group was injected with rAAV-hSyn-EGFP. NS refers to not significant. (C) Total distance traveled for HMGA2 gene knockout mice in the rotarod test. ko: ko group: Hmga2-/- mice, n=16. het group: heterozygous mice, n=8. wt group: wild type mice, n=9. *, $P < 0.05$.

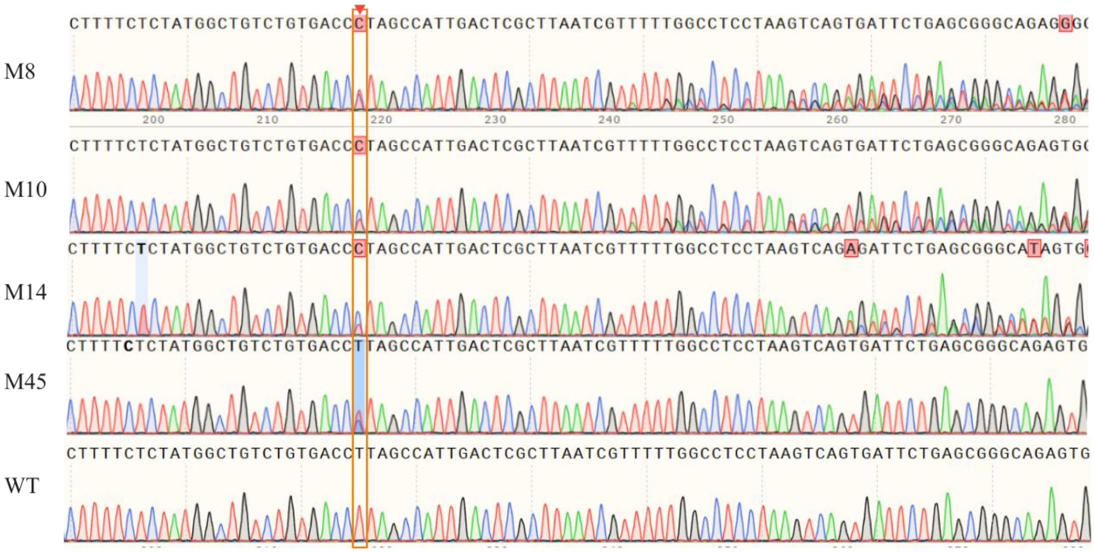

Figure S4. Results of sanger sequencing of clones, related to Figure 4. All these clones have heterozygous mutation at the target site. M8, M10, and M14 have an extra InDel.

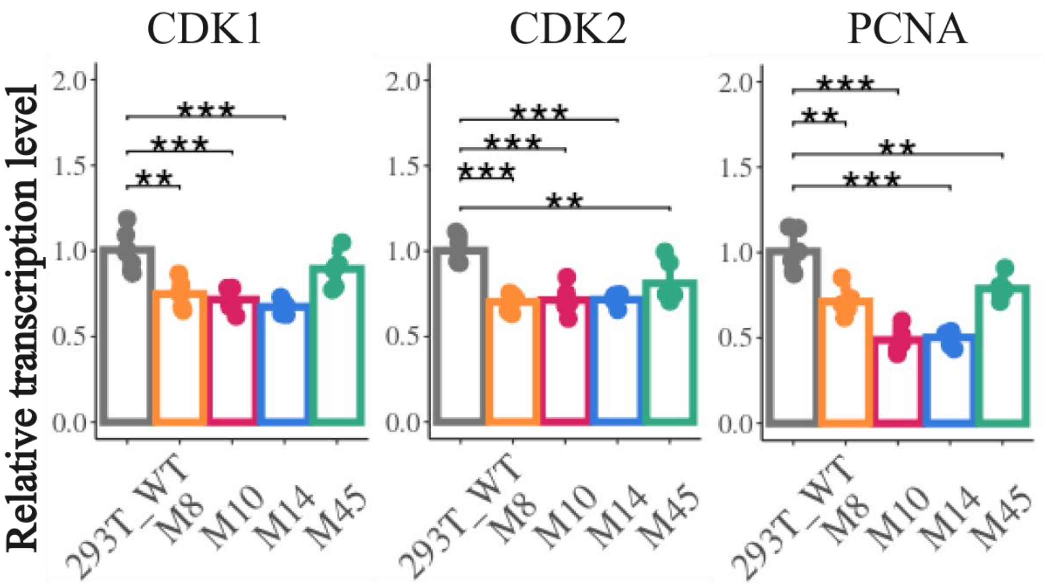

Figure S5. Relative mRNA level of marker genes (CDK1, CDK2 and PCNA) related to cell proliferation, related to Figure 4. M45 is cell line with heterozygous T>C mutation at the homologous site (hg38 chr12:65853604). M10, M14 and M8 cell lines have an extra InDel besides the heterozygous T>C mutation. **, $P < 0.01$; ***, $P < 0.001$.

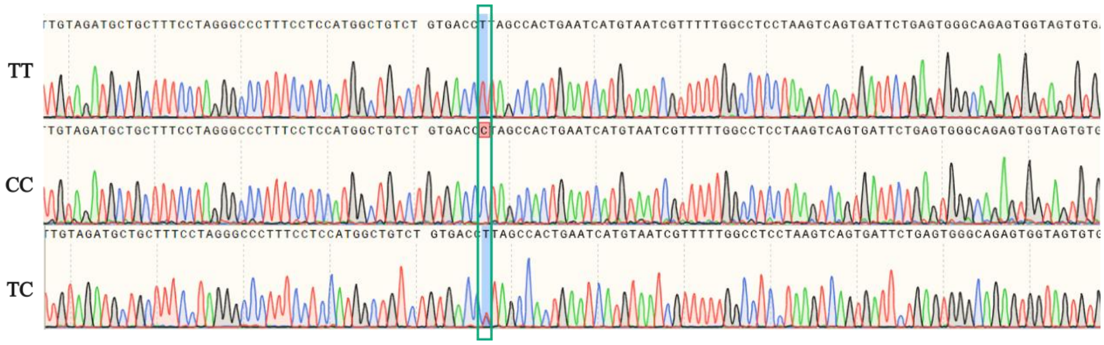

Figure S6. Results of sanger sequencing for genotype TT, CC, and TC, respectively. Related to Figure 4.
